# Exploring for HPLC-MS/MS Profiles and Biological Activities of Different Extracts from *Allium lycaonicum* Siehe ex Hayek from Turkey Flora

**DOI:** 10.3390/foods12244507

**Published:** 2023-12-17

**Authors:** Sakina Yagi, Nilofar, Gokhan Zengin, Evren Yildiztugay, Giovanni Caprioli, Diletta Piatti, Luigi Menghini, Claudio Ferrante, Simonetta Cristina Di Simone, Annalisa Chiavaroli, Filippo Maggi

**Affiliations:** 1 Institut National de Recherche pour l’Agriculture, l’Alimentation et l’Environnement, Laboratoire Agronomie Environnement, Université de Lorraine, 54000 Nancy, France; sakinayagi@gmail.com; 2 Department of Botany, Faculty of Science, University of Khartoum, Khartoum 11115, Sudan; 3 Physiology and Biochemistry Laboratory, Department of Biology, Science Faculty, Selcuk University, Konya 42130, Turkey; nilofar.nilofar@unich.it (N.); gokhanzengin@selcuk.edu.tr (G.Z.); 4 Department of Pharmacy, Botanic Garden “Giardino dei Semplici”, Università degli Studi “Gabriele d’Annunzio”, via dei Vestini 31, 66100 Chieti, Italy; luigi.menghini@unich.it (L.M.); claudio.ferrante@unich.it (C.F.); simonetta.disimone@unich.it (S.C.D.S.); 5 Department of Biotechnology, Science Faculty, Selcuk University, Konya 42130, Turkey; eytugay@gmail.com; 6 Chemistry Interdisciplinary Project (ChIP), School of Pharmacy, University of Camerino, Via Madonna delle Carceri 9/B, 62032 Camerino, Italy; giovanni.caprioli@unicam.it (G.C.); diletta.piatti@unicam.it (D.P.); filippo.maggi@unicam.it (F.M.)

**Keywords:** *Allium lycaonicum*, maceration, Soxhlet/infusion, phenolic constituents, antioxidant, enzyme inhibition

## Abstract

The present study was designed to determine the phenolic constituents, antioxidant, and enzyme inhibition activities of aerial parts and bulbs of *Allium lycaonicum* (family Amaryllidaceae). Extracts were prepared by maceration and Soxhlet/infusion using hexane, methanol, and water as extraction solvents. Generally, extracts from the aerial parts showed higher total phenolic and individual components and antioxidant activity than their respective bulb extracts. Maceration with water was the best to extract total phenolic content from the aerial parts (29.00 mg gallic acid equivalents (GAE)/g), while the Soxhlet extraction with hexane (22.29 mg GAE/g) was the best for the bulb. Maceration with methanol recovered the highest total flavonoid content from both the aerial parts (41.95 mg (rutin equivalents (RE)/g) and bulb (1.83 mg RE/g). Polar extracts of aerial parts were characterized by higher abundance of kaempferol-3-glucoside (≤20,624.27 µg/mg), hyperoside (≤19,722.76 µg/g), isoquercitrin (≤17,270.70 µg/g), delphindin-3,5-diglucoside (≤14,625.21 µg/g), and rutin (≤10,901.61 µg/g) than the bulb. Aerial parts’ aqueous extract, prepared by maceration, exerted the highest anti-ABTS (2,2′-azino-bis(3-ethylbenzothiazoline-6-sulfonic acid) radical activity (64.09 mg trolox equivalents (TE)/g), Cu^++^ (83.03 mg TE/g) and Fe^+++^ (63.03 mg TE/g) reducing capacity while that prepared by infusion recorded the highest anti-DPPH (2,2-diphenyl-1-picrylhydrazyl) radical (31.70 mg TE/g) and metal chelating (27.66 mg EDTAE/g) activities. The highest total antioxidant activity (1.46 mmol TE/g) was obtained by maceration of the bulb with water. Extracts obtained by organic solvents showed remarkable enzyme inhibition properties against the tested enzymes. Soxhlet extraction of the bulb with hexane and methanol recorded the highest acetylcholinesterase inhibition (4.75 mg galanthamine equivalents (GALAE)/g) and tyrosinase inhibition (139.95 mg kojic acid equivalents/g) activities, respectively. Extracts obtained by maceration of the bulb with methanol and the aerial parts with hexane exerted the highest glucosidase inhibition (3.25 mmol acarbose equivalents/g) and butyrylcholinesterase inhibition (20.99 mg GALAE/g) activities, respectively. These data indicated that *A. lycaonicum* is a source of bioactive molecules with potential antioxidant and enzyme inhibition properties. Nonetheless, the extracts obtained through various solvents and extraction techniques showed variations in their phytoconstituent composition and biological properties.

## 1. Introduction

Natural products from plants or microorganisms possess valuable curative benefits to humans and animals. They provide a continuing source of bioactive agents with a wide range of applications in the agricultural, food, medical, pharmaceutical, and cosmetics sectors. Consequently, much research has been devoted to exploring novel sources of extracts/molecules with potent biological activities like antioxidant, antidiabetics, antimicrobial, anti-inflammatory, anticancer, and antiaging [1,2]. However, bioactive molecules present in plants have diverse structures and polarities. Thus, different extraction solvents and techniques may have variable effects on the solubility, extraction yield, and biological activity of the extracts/compounds [3]. Accordingly, choosing appropriate solvents and extraction techniques depends on the plant materials’ nature and type and the intended biological testing [4]. 

The genus *Allium*, family Amaryllidaceae, comprises about 900 species, of which 230 are endogenous to Turkey [5]. The Mediterranean Basin to Central Asia and Pakistan are the main centers of diversity of the *Allium* species, in addition to a minor one found in western North America [6]. The genus includes many vegetable crops and wild species with valuable uses as food and spice, like *A. sativum* (garlic), *A. cepa* (onion), and *A. porrum* (leek). Many *Allium* species are also used for their health-beneficial properties to treat diseases like stomachache, diabetes, constipation, cold, cough, asthma, hypertension, and skin diseases and as anthelmintic [6,7]. Previous review articles indicated *Allium* species are rich in secondary metabolites like organosulfur compounds, phenols, flavonoids, alkaloids, saponins, sterols, and essential oils [8,9,10,11]. They also produced a broad spectrum of biological activities like antioxidant, antimicrobial, antiviral, anticancer, anti-inflammatory, and antidiabetic [8,12,13]. The species *A. lycaonicum* belongs to the section Decipientia and subgenus *Melanocrommyum* (Webb & Berthel.) Rouy. It is found in Turkey in central and western Anatolia [14,15]. *A. lycaonicum* is used as an ornamental plant and food source to cure coughs and colds. To the best of our knowledge and according to a literature search, the chemical constituents and biological activities of *A. lycaonicum* have not been reported elsewhere. 

The development of functional foods is critical to improving the quality of life and promoting better health. We hypothesized that *A. lycaonicum* could be a promising new source of bioactive phytoconstituents for developing functional food formulations. The present study was designed to determine the phenolic constituents, antioxidant, and enzyme inhibition activities of the aerial parts and bulb of *A. lycaonicum*, focusing on identifying the optimal extraction solvent and method for maximizing phenolic content and biological activity. Antioxidant activity was assessed by examining their capacity to scavenge radicals, reduce ions, and chelate metal. The enzyme inhibition properties were evaluated against acetylcholinesterase (AChE), butyrylcholinesterase (BChE), tyrosinase (Tyr), α-amylase, and α-glucosidase enzymes. Additionally, the relationship between phytoconstituents and the assessed biological activities was highlighted.

## 2. Materials and Methods

### 2.1. Reagents and Standards

Cyanidin-3-glucoside chloride, delphinidin-3,5-diglucoside chloride, delphinidin-3-galactoside chloride, petunidin-3-glucoside chloride, malvidin-3-galactoside chloride, quercetin-3-glucoside and kaempferol-3-glucoside were purchased from PhytoLab (Vestenbergsgreuth, Germany). The other 31 analytical standards of the 38 phenolic compounds were supplied by Sigma-Aldrich (Milan, Italy). Individual stock solutions of each analyte, at a concentration of 1000 mg L^−1^, were prepared by dissolving pure standard compounds in HPLC-grade methanol and storing them in glass stoppered bottles at 4 °C except anthocyanins, which were stored at −15 °C until analysis. Formic acid (99%) was obtained from Merck (Darmstadt, Germany). Analytical-grade hydrochloric acid (37%) was obtained from Carlo Erba Reagents (Milan, Italy). HPLC-grade methanol was supplied by Sigma-Aldrich (Milan, Italy). Deionized water (>18 MΩ cm resistivity) was further purified using a Milli-Q SP Reagent Water System (Millipore, Bedford, MA, USA). All solvents and solutions were filtered through a 0.2 μm polyamide filter from Sartorius Stedim (Goettingen, Germany). Before HPLC analysis, all samples were filtered with a Phenex™ RC 4 mm 0.2 μm syringeless filter, Phenomenex (Castel Maggiore, BO, Italy).

### 2.2. Plant Material

*Allium lycaonicum* was collected during the summer period of 2021 at the territory of Hadim (Çat location, 1560 m, Konya) in Turkey. Dr. Evren Yildiztugay identified and deposited plant material at the University of Selcuk. The aerial parts and bulbs of the plant were dried for ten days with airing, then crushed to powder form (by using Retsch, Haan, Germany, SM-200, almost particle size 2 mm) and stored in paper bags protected from moisture. 

### 2.3. Extraction Process

We used two extraction methods (maceration and Soxhlet) to obtain extracts using three solvents (*n*-hexane, methanol, and water). Our research involved using various polar solvents to analyze the impact on chemical compositions and biological effects [16,17]. In the maceration, the plant samples (aerial parts and bulbs) (10 g) were mixed with these solvents (200 mL) for 24 h at room temperature. The plant samples (10 g) were extracted in a Soxhlet apparatus for 6 h. Then, the extracts were filtered, and the solvents were removed using a rotary evaporator.

Regarding infusion, the plant materials (10 g) were kept in the boiled water (200 mL) for 15 min. Then, the extracts were filtered and lyophilized. The obtained extracts were stored at 4 °C until analysis. To analyze the extracts, the *n*-hexane and methanol extracts were dissolved in methanol, while the water extracts were dissolved in water. 

### 2.4. Phytochemical Screening

Total phenolic and flavonoid contents in the extracts were determined by Folin–Ciocalteu (F9252, Merck) and AlCl_3_ (11019, Merck) assays, respectively, and the procedures are reported in our earlier paper [18]. Experimental details are given in the Supplemental Materials.

### 2.5. HPLC-ESI-MS/MS Triple Quadrupole 

**Figure 1:**
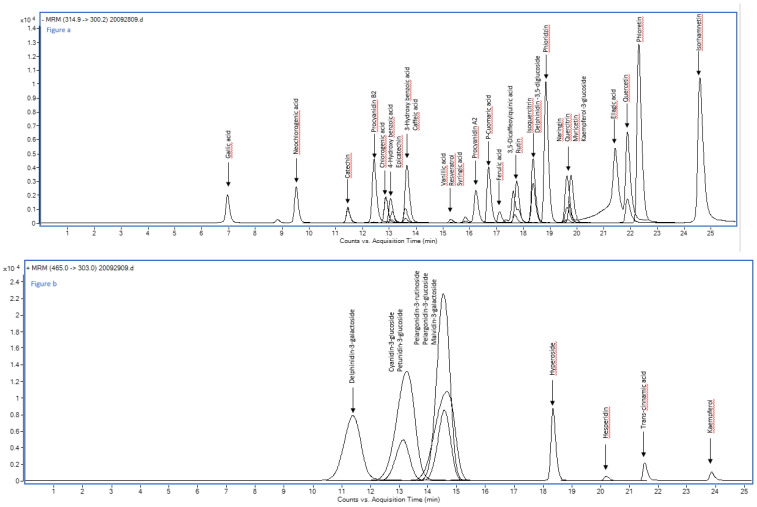
HPLC-MS/MS chromatogram of a standard mixture of 38 phenolic compounds plotted as overlapped multiple reaction monitoring (MRM) negative (**a**) and positive (**b**) transition of each analyte.

HPLC-MS/MS studies were performed using an Agilent 1290 Infinity series and a Triple Quadrupole 6420 from Agilent Technology (Santa Clara, CA, USA) equipped with an electrospray ionization (ESI) source operating in negative and positive ionization modes. The instrument allowed a single run to be performed with polarity switching without any problems. MS/MS parameters of each analyte were optimized in flow injection analysis (FIA) (1 μL of a 10 mg L^−1^ individual standard solution) by using Optimizer Software (Version 5, Agilent). The separation of target compounds was achieved on a Synergi Polar–RP C18 analytical column (250 mm × 4.6 mm, 4 µm) from Phenomenex (Chesire, UK). The column was preceded by a Polar RP security guard cartridge (4 mm × 3 mm ID). The mobile phase was a mixture of (A) water and (B) methanol, both with formic acid (0.1%), at a flow rate of 0.8 mL min^−1^ in gradient elution mode. The composition of the mobile phase varied as follows: 0–1 min, isocratic condition, 20% B; 1–25 min, 20–85% B; 25–26 min, isocratic condition, 85% B; 26–32 min, 85–20% B. Before HPLC analysis, freeze-dried samples were dissolved in methanol and filtered through a 0.2 μm polyamide filter from Sartorius Stedim (Goettingen, Germany). The injection volume was 2 μL. The column temperature was 30 °C, and the drying gas temperature in the ionization source was 350 °C. The gas flow was 12 L/min, the nebulizer pressure was 55 psi, and the capillary voltage was 4000 V. Detection was performed in the dynamic-multiple reaction monitoring (dynamic-MRM) mode. The dynamic-MRM peak areas were integrated for quantification. The most abundant product ion was used for quantitation and the others for qualification. The specific time window for each compound (Δ retention time) was set at 2 min. The selected ion transitions and the mass spectrometer parameters for the analyzed compounds are reported in Table S1. The chromatogram for standard compounds is given in Figure 1.

### 2.6. HPLC-ESI-MS/MS Method Validation

The analytical method was validated in terms of linearity, limits of detection (LODs), quantification (LOQs), repeatability, and specificity. Calibration curves were constructed by injecting standard mixture solutions at the eight concentrations of 0.001, 0.005, 0.01, 0.05, 0.1, 0.5, 1, and 5 mg/L, and all analytes showed good linearity (R^2^ ≥ 0.9958). The LODs and LOQs were obtained by injecting serial dilutions of the corresponding standard solutions, taking the signal-to-noise (S/N) ratio of 3 and 10 as criteria, respectively. The signal-to-noise (SNR) ratio was measured using MassHunter Software (Version 5.1) from Agilent Technology (Santa Clara, CA, USA). The LODs ranged from 0.0004 to 0.0033 mg/L, while the LOQs ranged from 0.0012 to 0.01 mg/L, indicating good sensitivity. The intraday precision of the HPLC-MS/MS method was evaluated by injecting three times the analytical standards at a concentration of 0.1 mg/L. The interaday precision was evaluated by injecting the same concentration of analytical standards for three days. Precision measurements were expressed as relative standard deviations (RSDs). The method revealed good precision with inter- and intraday variations where RSD (%) ranged from 0.45 to 4.82 and 2.1 to 5.05, respectively. The method specificity was evaluated by measuring the stability of retention time three times for 5 days and expressed by RSDs %, which were in all cases ≤ 1.91%

### 2.7. Antioxidant and Enzyme Inhibition Assays

The antioxidant assays included the 2,2-diphenyl-1-picrylhydrazyl (DPPH, D9132, Merck), 2,2′-azino-bis(3-ethylbenzothiazoline-6-sulfonic acid (ABTS, A3219, Merck) assays, which examines the antioxidants’ ability to neutralize free radicals, ferric reducing antioxidant power (FRAP), Cupric reducing antioxidant capacity (CUPRAC) assays, which evaluate the extract’s reduction capabilities, as well as the metal chelating ability (MCA) and phosphomolybdenum (PBD) assays. Each assay was assessed using the trolox (238813, Merck) standard except for MCA. The comparison for MCA was made in terms of equivalent EDTA (798681, Merck) equivalent per gram of extract. All used procedures as given in our previous work by Nedić, et al. [19]. For enzyme inhibition, we employed acetylcholinesterase (AChE, from *Electrophorus electricus*, Type-VI-S, EC 3.1.1.7, Merck), butyrylcholinesterase (BChE, from horse serum, EC 3.1.1.8, Merck), tyrosinase, amylase (from ex-porcine pancreas, EC 3.2.1.1, Merck), and glucosidase (from *Saccharomyces cerevisiae*, EC 3.2.1.20, Merck). The levels of AChE and BChE inhibition were calculated as milligrams of galanthamine (G1660, Merck) equivalents (GALAE) per gram of extract, tyrosinase inhibition as milligrams of kojic acid (K3125, Merck) equivalents (KAE) per gram of extract, and α-amylase and α-glucosidase inhibition as millimoles of acarbose equivalents (A8980, Merck) (ACAE) per gram of extract. These measurements provide a standardized assessment of the inhibitory potential of the extracts on these enzymes Nedić, et al. [19]. Experimental details are given in the Supplemental Materials.

### 2.8. Statistical Analysis

Statistical analysis was conducted with the help of Xl Stat (Version 16, Addinsoft Inc., New York, NY, USA). Results were expressed as mean ± SD of 3 measurements. A significance level of *p* < 0.05 was applied to one-way ANOVA followed by Tukey’s post hoc test to identify statistically significant differences between groups. Using the Pearson correlation test, we assessed the link between phytoconstituents in the tested extracts and their corresponding biological activities. The correlation analysis was performed using GraphPad (GraphPad Software, version 9, San Diego, CA, USA).

## 3. Results and Discussion

The study involved the analysis of phytoconstituents, antioxidant activity, and enzyme inhibition in various extracts obtained from both the aerial parts (A) and the bulb (B) of *A*. *lycaonicum*. These extracts were prepared through two different methods: maceration (M) and hot extraction (soxhlet/infusion) (H). Each extract was designated by a specific code: For aerial parts obtained through maceration, we have MAH, MAM, and MAW, representing hexane, methanol, and water extracts, respectively. The codes for bulb extracts obtained via maceration are MBH, MBM, and MBW, signifying hexane, methanol, and water extracts, respectively. The aerial parts obtained using the hot extraction method have codes HAH, HAM, and HAW, corresponding to hexane, methanol, and water extracts; for bulb extracts obtained through hot extraction, the codes are HBH, HBM, and HBW, denoting hexane, methanol, and water extracts, respectively.

### 3.1. The Total Phenolic Content (TPC) and Flavonoid Content (TFC) 

Phenolic compounds and flavonoids are among the well-known classes of metabolites that possess interesting biological activities. In the present study, extracts obtained by maceration and hot extraction of the aerial parts and bulb of *A. lycaonicum* were examined for their TPC and TFC, and the results are depicted in Table 1. Extracts from the aerial parts contained higher TPC and TFC than their respective bulb extracts except the TPC of HBH extract. Maceration of aerial parts significantly (*p* < 0.05) recovered higher TPC content than the hot extraction, and it was in the following descending order; MAW (29.00 mg GAE/g) > MAH (21.83 mg GAE/g) > HAW (20.84 mg GAE/g) > MAM (18.08 mg GAE/g) > HAM (17.75 mg GAE/g) > HAH (14.80 mg GAE/g). On the other hand, the extraction technique employed did not significantly impact the recovery of TPC from the bulb. However, when hexane was used as the solvent, it resulted in the highest TPC extraction (HBH = 22.29 mg GAE/g and MBH = 18.39 mg GAE/g, *p* < 0.05). On the other hand, the extraction technique did not remarkably affect the recovery of TPC from the bulb. However, *n*-hexane as a solvent extracted the highest TPC (HBH = 22.29 mg GAE/g and MBH = 18.39 mg GAE/g, *p* < 0.05). Other bulbs’ extracts recorded TPC ≤ 10.52 mg GAE/g. Concerning the TFC in aerial parts, the highest amount was obtained from the methanolic extracts (MAM = 41.95 mg RE/g and HAM = 32.16 mg RE/g). Moreover, infusion by water (HAW) extract yielded a 2.3-fold higher TFC than maceration with water (MAW). Maceration of the bulb with organic solvent recovered significantly (*p* < 0.05) higher TFC (MBM = 1.83 mg RE/g; MBH = 1.24 mg RE/g) than using the Soxhlet technique (HBM = 0.59 mg RE/g; HBH = 0.33 mg RE/g). Overall, it was observed that maceration and water as solvents were the best to extract TPC from the aerial parts, followed by infusion with water. Meanwhile, for the recovery of TFC, maceration followed by Soxhlet extraction using methanol in both methods demonstrated the highest efficacy. For the bulb hot and cold extraction, hexane was the best for the highest recovery of TPC and maceration with methanol for TFC. However, values of TPC were lower than those reported for other species like *A. scorodoprasum* (TPC of bulb = 36.692 mg GAE/g) [20], *A. scorodoprasum* subsp. *rotundum* (TPC of bulb = 21.78 and stem = 26.28 mg GAE/g) [21], and *A. stylosum* (TPC of bulb = 12.31 mg GAE/g) [22]. Regarding TFC, the obtained results were higher than those of earlier studies (0.23 mg RE/g for bulb of *A. scordoprasum* [20]; 0.75 mg RE/g for bulb of *A. scorodoprasum* subsp. *rotundum* [21]). These differences can be related to species-specific phenolic compositions and extraction protocols [23].

There have been serious concerns regarding spectrophotometric studies of total phenolics and flavonoid content in recent years [24,25,26]. First, in the Folin-Ciocalteu assay, phenolic and non-phenolic compounds, such as peptides, can react with the Folin-Ciocalteu reagent. This case may produce more than expected results [25,27]. In addition to the Folin-Ciocalteu assay, the AlCl_3_ assay has some disadvantages. For example, flavonoids are a large group, and several flavonoids have different values of absorption maxima and different absorption maxima, and most of them cannot form a complex with Al(III). For example, the relative absorption of quercetin at the absorption maxima is twice that of rutin at its absorption maxima [26]. From this point on, the quantification of flavonoids by AlCl_3_ depends directly on the standards used [26,28,29]. For example, if a sample contains no or only small amounts of catechin, using quercetin or rutin as standards will produce incorrect results [26]. In this sense, as shown in Table 1, the TFC value of the methanolic extract of the aerial part was higher than that of TPC. The unexpected results can be explained by the reasons mentioned above. Overall, the results gathered from spectrophotometric research need to be confirmed using chromatographic techniques like HPLC or LC-MS/MS.

### 3.2. Phytoconstituents Profile

**Figure 2:**
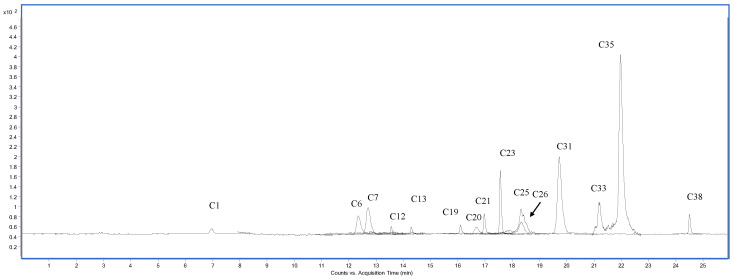
HPLC-MS/MS chromatogram of the water extract (HAW indicated in Table 2) of aerial parts plotted as overlapped multiple reaction monitoring (MRM) transition of each analyte in negative polarity. (C1) Gallic acid, (C6) chlorogenic acid, (C7) *p*-hydroxybenzoic acid, (C12) caffeic acid, (C13) vanillic acid, (C19) procyanidin A2, (C20) *p*-coumaric acid, (C21) ferulic acid, (C23) rutin, (C25) isoquercitrin, (C26) delphindin-3,5-diglucoside, (C31) kaempferol-3-glucoside, (C33) ellagic acid, (C35) quercetin, (C38) isorhamnetin.

**Figure 3:**
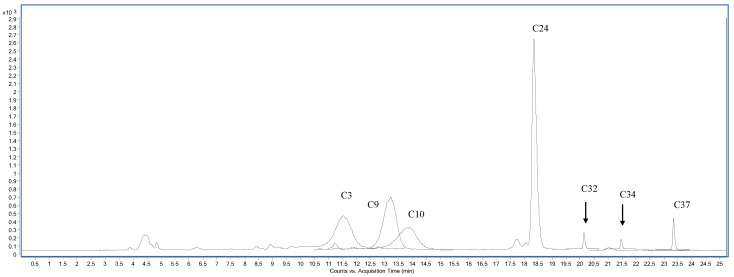
HPLC-MS/MS chromatogram of the water extract (HAW indicated in Table 2) of aerial parts sample plotted as overlapped multiple reaction monitoring (MRM), transition of each analyte in positive polarity. (C3) Delphindin-3-galactoside, (C9) cyanidin-3-glucoside, (C10) petunidin-3-glucoside, (C24) hyperoside, (C32) hesperidin, (C34) trans-cinnamic acid, (C37) kaempferol.

The phenolic profile of extracts was determined using 38 standards, and results are presented in Table 2. The distribution and concentrations of detected compounds varied according to the plant organ, technique of extraction, and solvent used. The sample chromatograms (in negative and positive polarity) of HAW are shown in Figure 2 and Figure 3. Extracts of aerial parts showed higher total bioactive content than their respective bulb extracts. The two polar extracts of aerial parts obtained by Soxhlet (HAM) and infusion (HAW), in addition to the MAM extract obtained by maceration, recovered the highest total bioactive individuals (87,913.60, 87,704.17, and 84,958.57 µg/g, respectively). In contrast, the hexane extracted the least content (164.00–4202.23 µg/g). However, hot extraction of the bulbs recovered higher total bioactive individuals (3330.35–41,782.56 µg/mg) than the maceration (898.42–3258.81 µg/g) method. Although the hexane extracts of aerial parts recorded the least total bioactive content, the hexane extract of the bulb obtained by the Soxhlet technique revealed the highest total bioactive individuals (41,782.56 µg/g). The three polar extracts of aerial parts (HAM, HAW, and MAM) were characterized by the abundance of kaempferol-3-glucoside (20,421.73–20,624.27 µg/g), kaempferol (1574.37–2243.51 µg/g), hyperoside (16,802.51–19,722.76 µg/g), isoquercitrin (16,105.47–17,270.70 µg/g), delphindin-3,5-diglucoside (13,301.42–14,625.21 µg/g), *p*-hydroxybenzoic acid (929.66–1274.64 µg/g), rutin (9448.38–10,901.61 µg/g), quercetin (770.12–872.12 µg/g), ellagic acid (701.53–703.94 µg/g), *p*-coumaric acid (298.37–343.62 µg/g), ferulic acid (279.45–313.69 µg/g), trans-cinnamic acid (201.76–232.66 µg/g), isorhamnetin (107.30–147.32 µg/g), and delphindin-3-galactoside (115.28–161.92 µg/g). The aqueous extract obtained by maceration (MAW) accumulated the highest content of *p*-hydroxybenzoic acid (9224.09 µg/g), vanillic acid (1987.28 µg/g), and gallic acid (101.07 µg/g). Although the hexane extracts of aerial parts displayed the least number and concentration of bioactive compounds, MAH had considerable amounts of kaempferol-3-glucoside (1202.95 µg/g). Concerning the bulb extracts, most of the aforementioned compounds were also detected in the three bulb extracts prepared by hot extraction but in lower concentrations. Neochlorogenic acid (MBH = 277.40 µg/g) and chlorogenic acid (HBM = 20.85 µg/g and HBW = 20.73 µg/g) were mainly identified on bulb extracts. Additionally, the three polar extracts of the bulb, HBM, HBW, and MBM, displayed higher amounts of caffeic acid (204.73, 274.16, and 275.22 µg/g, respectively) compared to aerial parts extracts. These results agreed with previous reports stating that extraction solvent and technique influence plant extracts’ phytochemical profile (in terms of quality and quantity) [3,4]. Variations in the profile and quantity of phytoconstituents in aerial parts and bulbs may be related to their different functions in the two organs [30]. Most of these phytoconstituents were also identified in many *Allium* species [8,9,10,11]. 

### 3.3. Antioxidant Activity

**Figure 4:**
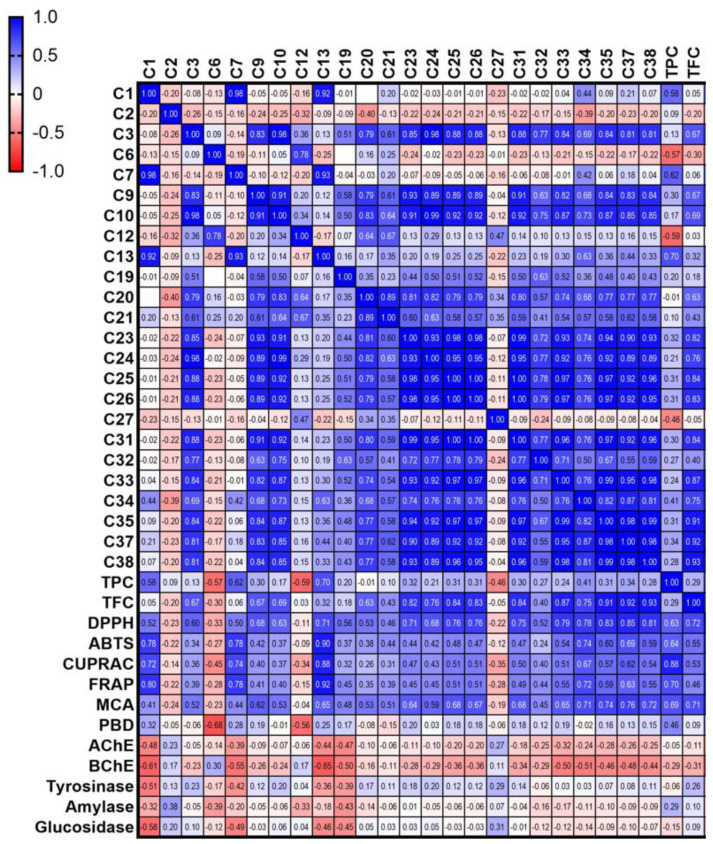
Correlation analysis of the phytochemical composition and biological activities. ABTS,2,2′-azino-bis (3-ethylbenzothiazoline) 6-sulfonic acid. AChE: acetylcholinesterase; BChE: butyrylcholinesterase; CUPRAC: cupric ion reducing antioxidant capacity; DPPH: 1,1-diphenyl-2-picrylhydrazyl; FRAP: ferric ion reducing antioxidant power; MCA: metal chelating activity; PBD: phosphomolybdenum activity; TPC: total phenolic content; TFC: total flavonoid content. Compounds numbered as in Table 2.

Antioxidants play a significant protective effect against free radical attacks, contributing to serious health problems. To provide an account of the antioxidant potential of the aerial parts and bulb of *A. lycaonicum* from different perspectives, six complementary assays, including measuring the antiradical (DPPH and ABTS assays), electron donation (CUPRAC and FRAP assays), transformation of Mo(VI) to Mo(V) under acidic conditions (phosphomolybdenum assay), and metal chelating properties were performed. Results are presented in Table 3. The capacity of most extracts of the aerial parts to scavenge the DPPH and ATBS radicals was higher than their respective bulb extracts, with higher values recorded in the ABTS assay. The highest significant (*p* < 0.05) anti-DPPH radical was exhibited by HAW (31.70 mg TE/g), followed by MAM and MAW, which exerted comparable values (28.00 and 26.81 mg TE/g, *p* > 0.05) and HAM (22.50 mg TE/g). In the ABTS assay, the highest antiradical activity was displayed by MAW (64.09 mg TE/g). MAM (43.89 mg TE/g) and HAW (43.77 mg TE/g) revealed comparable capacity (*p* > 0.05), while HAM recorded a value of 27.26 mg TE/g. In both assays, the hexane extracts had the least activity. Except for HAH, all extracts from the aerial parts showed a higher capacity to reduce the Cu^2+^ and Fe^3+^ ions than their respective bulb extracts, with a higher ability to reduce the former ion. The MAW extract exhibited the highest significant (*p* < 0.05) capacity for reducing Cu^2+^ (83.03 mg TE/g) and Fe^3+^ (63.03 mg TE/g) ions, followed by HAW (61.90 mg TE/g and 51.33 mg TE/g, respectively). Also, the bulb’s hexane extract (HBH) displayed considerable Cu^2+^ reducing ability (41.62 mg TE/g). On the other hand, the bulb extracts MBW (1.46 mmol TE/g), and HBH (1.11 mmol TE/g) exhibited the highest ability to reduce Mo. Three aerial parts extracts, HAW (27.66 mg EDTAE/g), MAM (25.25 mg EDTAE/g), and MAW (24.57 mg EDTAE/g), recorded the highest ability to chelate the iron metal (*p* > 0.05), followed by MAH (20.68 mg EDTAE/g) and HAM (20.11 mg EDTAE/g). Overall, it was observed that the aerial parts with aqueous extracts prepared by maceration or infusion exerted the highest antioxidant activity in most assays, which was consistent with their higher TPC. Among the identified compounds, kaempferol, quercetin, gallic acid, *p*- hydroxybenzoic acid, 3-hydroxybenzoic acid, caffeic acid, ferulic acid, vanillic acid, *p*-coumaric acid, ellagic acid, rutin, and hyperoside are suggested to participate in the observed antioxidant activity of different extracts [31,32,33,34,35,36]. This fact was also confirmed by correlation analysis, and the results are shown in Figure 4. Apparently, some compounds (gallic acid, cyanidin-3-glucoside, vanillic acid, trans-cinnamic acid, kaempferol, and isorhamnetin) were strongly correlated with the radical scavenging and reducing abilities of the tested extracts. Furthermore, these results were consistent with previous reports indicating that the *Allium* species are a promising source of antioxidant agents [20,21,37,38,39,40,41]. 

### 3.4. Enzyme Inhibition Activity

Inhibitors from plants are known as an essential approach to treating neurodegenerative diseases and many non-communicable diseases like Alzheimer’s disease and diabetes. The enzyme inhibition properties of aerial parts and bulb extracts of *A. lycaonicum* were evaluated against AChE, BChE, Tyr, α-amylase, and α-glucosidase enzymes. Results are presented in Table 4. All organic extracts from both the aerial parts and the bulb revealed potent anti-AChE activity with the highest and comparable values (4.59–4.20 mg GALAE/g, *p* > 0.05) recorded from HBH, HBM, MAH, MBM, MBH,3 and HAH. This was followed by MAM (3.27 mg GALAE/g) and HAM (3.08 mg GALAE/g). Additionally, all organic extracts, in addition to the aqueous extract of the bulb obtained by infusion (HBW), exerted remarkable anti-BChE activity in the range of 12.28–20.99 mg GALAE/g with MAH and HAM recording the highest and lowest effect, respectively. It was also noted that extracts showed higher activity against the BChE enzyme than the AChE one, consistent with the findings of Karakaya et al. [42] and Amir and Emir [22] against *A. tuncelianum* and *A. stylosum*, respectively. Furthermore, data in the literature suggested that the cholinesterase inhibition property of the *Allium* species can be attributed to various classes of metabolites, mainly sulfur compounds and phenolics, and their synergistic effects [23,43]. However, among the identified compounds in the present study, kaempferol, quercetin, and rutin were demonstrated for their cholinesterase inhibition activity [44,45]. As can be seen in Figure 4, we did not observe any correlation between individual/total compounds and the tested enzymes. Both methanol extracts (MAM and HAM) of the aerial parts, as well as HBM from the bulb, had the highest anti-Tyr activity (138.95–139.95 mg KAE/g, *p* > 0.05) followed by MBM (132.39 mg KAE/g), HAH (129.86 mg KAE/g), MAH (127.35 mg KAE/g), HBH (125.30 mg KAE/g) and MBH (118.67 mg KAE/g), respectively. All aqueous extracts showed a low anti-Tyr effect. The anti-Tyr capacity of some *Allium* species, like *A. thunbergi* and *A. tuberosum* [46], *A. ursinum* [47], and *A. stylosum* [22], has also been demonstrated, further supporting the current findings. Inhibition of α-amylase and α-glucosidase enzymes delays the breakdown of carbohydrates, reducing the postprandial blood glucose level. In the present study, hexane extracts of both the aerial parts and the bulb showed considerable α-amylase inhibition activity (0.62–0.73 mmol ACAE/g), with MBH and HAH recording the highest and lowest values, respectively. All organic extracts of the two studied parts showed high α-glucosidase inhibition activity (2.53–3.25 mmol ACAE/g) with MBM and MAM revealing the highest and lowest values, respectively. Previous studies demonstrated that quercetin is one of the main compounds responsible for the α-glucosidase and α-amylase inhibition in *A. cepa* [48]. 

## 4. Conclusions

The present study represented the first report on the phytoconstituents and biological activity of *A. lycaonicum.* Results indicated that the total phenolics, distribution, and concentrations of detected compounds and antioxidant and enzyme inhibition activities varied according to the plant organ, technique of extraction, and solvent used. Given these findings, *A. lycaonicum* could be a promising source of bioactive molecules with significant antioxidant and enzyme inhibition properties that can have diverse applications. Additionally, aqueous extracts can be considered an effective and cheap source of antioxidant agents from aerial parts, while hexane or methanol, through cold or hot extraction, can recover substances from both the aerial parts and bulb, with significant enzyme inhibition properties. Further understanding of these biological activities in in vivo models is needed. More detailed studies on the mechanisms of action of extracts would be beneficial to achieve their potential health benefits. It is also worth investigating the contribution of these extracts to other possible pharmacological attributes.

## Figures and Tables

**Table 1:** Total phenolic and flavonoid contents in extracts from aerial parts and bulb of *Allium lycaonicum*.

Extraction Methods	Parts	Solvents	Codes	Total Phenolic Content (mg GAE/g)	Total Flavonoid Content (mg RE/g)
Maceration	Aerial parts	*n*-Hexane	MAH	21.83 ± 0.45 ^bc^	9.18 ± 0.96 ^d^
		MeOH	MAM	18.08 ± 0.36 ^d^	41.95 ± 0.84 ^a^
		Water	MAW	29.00 ± 0.30 ^a^	7.64 ± 1.07 ^d^
	Bulb	*n*-Hexane	MBH	18.39 ± 0.22 ^d^	1.24 ± 0.07 ^f^
		MeOH	MBM	7.86 ± 0.09 ^gh^	1.83 ± 0.43 ^e^
		Water	MBW	10.52 ± 0.27 ^f^	0.42 ± 0.03 ^i^
Soxhlet/Infusion *	Aerial parts	*n*-Hexane	HAH	14.80 ± 0.31 ^e^	6.73 ± 0.72 ^d^
		MeOH	HAM	17.75 ± 0.70 ^d^	32.16 ± 1.53 ^b^
		Water	HAW	20.84 ± 0.40 ^c^	17.90 ± 1.80 ^c^
	Bulb	*n*-Hexane	HBH	22.29 ± 0.37 ^b^	0.33 ± 0.01 ^k^
		MeOH	HBM	8.39 ± 0.11 ^g^	0.59 ± 0.05 ^h^
		Water	HBW	7.25 ± 0.05 ^h^	0.76 ± 0.10 ^g^

* infusion method used to prepare water extracts; values are reported as mean ± SD of three parallel measurements. GAE: Gallic acid equivalents; RE: Rutin equivalents. Different letters indicate significant differences in the tested extracts (*p* < 0.05).

**Table 2:** Chemical composition (µg/g extract) of extracts from aerial parts and bulb of *Allium lycaonicum*.

Compounds	Maceration						Soxhlet/Infusion					
	Aerial Parts			Bulb			Aerial Parts			Bulb		
	*n*-Hexane	MeOH	Water	*n*-Hexane	MeOH	Water	*n*-Hexane	MeOH	Water	*n*-Hexane	MeOH	Water
	MAH	MAM	MAW	MBH	MBM	MBW	HAH	HAM	HAW	HBH	HBM	HBW
Gallic acid (C1)	nd	16.71	101.07	nd	nd	22.88	nd	17.16	17.16	17.61	10.28	10.28
Neochlorogenic acid (C2)	nd	nd	nd	277.40	nd	nd	nd	nd	nd	nd	nd	nd
Delphindin-3-galactoside (C3)	17.47	115.28	nd	nd	4.84	nd	nd	161.92	158.67	68.82	108.75	5.39
(+)-Catechin (C4)	nd	nd	nd	nd	nd	nd	nd	nd	nd	nd	nd	nd
Procyanidin B2 (C5)	nd	nd	nd	nd	nd	nd	nd	nd	nd	nd	nd	nd
Chlorogenic acid (C6)	nd	nd	nd	nd	nd	nd	nd	nd	3.67	nd	20.85	20.73
*p*-Hydroxybenzoic acid (C7)	221.35	1274.64	9224.09	nd	318.66	819.68	nd	964.95	929.66	937.04	296.62	296.92
(−)-Epicatechin (C8)	nd	nd	nd	nd	nd	nd	nd	nd	nd	nd	nd	nd
Cyanidin-3-glucoside (C9)	53.14	503.21	8.87	nd	26.85	nd	nd	239.39	524.16	351.08	130.46	24.08
Petunidin-3-glucoside (C10)	nd	15.15	nd	nd	nd	nd	nd	16.18	17.64	9.88	10.32	0.97
3-Hydroxybenzoic acid (C11)	nd	nd	nd	nd	nd	nd	nd	nd	nd	nd	nd	nd
Caffeic acid (C12)	nd	134.27	34.31	nd	204.73	nd	nd	126.28	127.71	76.52	274.16	275.22
Vanillic acid (C13)	nd	485.33	1987.28	159.28	nd	nd	nd	670.27	631.78	79.11	nd	nd
Resveratrol (C14)	nd	nd	nd	nd	nd	nd	nd	nd	nd	nd	nd	nd
Pelargonidin-3-glucoside (C15)	nd	nd	nd	nd	nd	nd	nd	nd	nd	nd	nd	nd
Pelagonidin-3-rutinoside (C16)	nd	nd	nd	nd	nd	nd	nd	nd	nd	nd	nd	nd
Malvidin-3-galactoside (C17)	nd	nd	nd	nd	nd	nd	nd	nd	nd	nd	nd	nd
Syringic acid (C18)	nd	nd	nd	nd	nd	nd	nd	nd	nd	nd	nd	nd
Procyanidin A2 (C19)	nd	nd	nd	nd	nd	nd	nd	nd	50.10	nd	nd	nd
*p*-Coumaric acid (C20)	43.13	343.62	101.88	nd	224.54	nd	nd	298.37	299.59	221.19	194.59	187.05
Ferulic acid (C21)	106.26	313.69	243.88	170.54	264.43	38.66	41.75	279.45	279.67	259.79	252.50	253.16
3,5-Dicaffeoylquinic acid (C22)	nd	nd	nd	nd	nd	nd	nd	nd	nd	nd	nd	nd
Rutin (C23)	537.19	10,901.61	187.26	22.34	422.55	nd	nd	9472.57	9448.38	6655.01	386.01	387.80
Hyperoside (C24)	781.99	16,802.51	718.77	84.32	398.51	nd	nd	19,722.76	19,400.64	9183.12	9188.06	449.85
Isoquercitrin (C25)	684.85	16,105.47	508.57	36.78	276.02	nd	nd	17,270.70	17,055.02	7243.23	307.02	309.15
Delphindin-3,5-diglucoside (C26)	553.91	13,301.42	410.92	27.54	258.41	nd	nd	14,718.52	14,625.21	6184.17	290.30	281.06
Phloridzin (C27)	nd	4.47	nd	nd	14.97	nd	nd	nd	nd	1.48	2.15	2.18
Quercitrin (C28)	nd	nd	nd	nd	nd	nd	nd	nd	nd	nd	nd	nd
Myricetin (C29)	nd	nd	nd	nd	nd	nd	nd	nd	nd	nd	nd	nd
Naringin (C30)	nd	nd	nd	nd	nd	nd	nd	nd	nd	nd	nd	nd
Kaempferol-3-glucoside (C31)	1202.95	20,441.65	501.94	29.81	726.49	nd	nd	20,421.73	20,624.27	9691.86	681.56	665.07
Hesperidin (C32)	nd	nd	nd	nd	nd	nd	nd	151.33	153.80	77.77	nd	12.59
Ellagic acid (C33)	nd	703.94	90.28	89.83	57.34	132.24	nd	703.96	701.53	132.73	54.56	52.83
trans-cinnamic acid (C34)	nd	232.66	191.13	nd	55.85	15.83	122.24	201.76	203.72	97.75	99.18	40.17
Quercetin (C35)	nd	872.12	141.69	nd	nd	nd	nd	780.43	770.12	138.19	12.70	11.60
Phloretin (C36)	nd	nd	nd	nd	nd	nd	nd	nd	nd	nd	nd	nd
Kaempferol (C37)	nd	2243.51	675.73	nd	nd	nd	nd	1585.54	1574.37	336.38	331.54	40.25
Isorhamnetin (C38)	nd	147.32	19.66	0.58	4.62	2.31	nd	110.34	107.30	19.84	4.02	3.99
Total	4202.23	84,958.57	15,147.33	898.42	3258.81	1031.60	164.00	87,913.60	87,704.17	41,782.56	12,655.63	3330.35

nd: not detected.

**Table 3:** Antioxidant properties of extracts from aerial parts and bulb of *Allium lycaonicum*.

Extraction Methods	Plant’s Parts	Solvents	Codes	DPPH (mg TE/g)	ABTS (mg TE/g)	CUPRAC (mg TE/g)	FRAP (mg TE/g)	PBD (mmol TE/g)	MCA (mg EDTAE/g)
Maceration	Aerial parts	*n*-Hexane	MAH	14.58 ± 1.07 ^d^	15.07 ± 1.90 ^d^	48.80 ± 1.49 ^d^	23.65 ± 0.88 ^f^	1.05 ± 0.19 ^bc^	20.68 ± 0.46 ^b^
		MeOH	MAM	28.00 ± 2.98 ^b^	43.89 ± 1.77 ^b^	54.11 ± 3.98 ^c^	36.63 ± 1.22 ^d^	0.95 ± 0.14 ^bcd^	25.25 ± 1.21 ^a^
		Water	MAW	26.81 ± 1.60 ^b^	64.09 ± 0.75 ^a^	83.03 ± 1.19 ^a^	63.03 ± 0.24 ^a^	1.06 ± 0.06 ^bc^	24.57 ± 1.94 ^a^
	Bulb	*n*-Hexane	MBH	9.08 ± 0.85 ^ef^	8.57 ± 0.34 ^fg^	34.05 ± 1.51 ^f^	19.13 ± 0.77 ^g^	0.80 ± 0.08 ^cd^	7.96 ± 0.70 ^d^
		MeOH	MBM	8.30 ± 0.47 ^f^	12.94 ± 0.55 ^de^	23.24 ± 0.52 ^hi^	18.34 ± 0.13 ^g^	0.88 ± 0.10 ^bcd^	8.01 ± 1.22 ^d^
		Water	MBW	12.96 ± 0.41 ^d^	14.69 ± 1.11 ^de^	29.65 ± 0.15 ^g^	26.32 ± 0.53 ^e^	1.46 ± 0.09 ^a^	2.13 ± 0.36 ^e^
Soxhlet/infusion *	Aerial parts	*n*-Hexane	HAH	6.21 ± 0.91 ^f^	4.75 ± 1.10 ^h^	29.16 ± 0.88 ^g^	16.13 ± 0.32 ^h^	0.41 ± 0.03 ^e^	8.30 ± 0.54 ^cd^
		MeOH	HAM	22.50 ± 0.054 ^c^	27.26 ± 1.71 ^c^	58.23 ± 0.46 ^bc^	39.89 ± 0.94 ^c^	0.75 ± 0.14 ^d^	20.11 ± 0.69 ^b^
		Water	HAW	31.70 ± 0.86 ^a^	43.77 ± 0.40 ^b^	61.90 ± 0.65 ^b^	51.33 ± 0.78 ^b^	1.04 ± 0.04 ^bcd^	27.66 ± 1.28 ^a^
	Bulb	*n*-Hexane	HBH	12.10 ± 0.47 ^de^	9.16 ± 1.00 ^fg^	41.62 ± 0.36 ^e^	24.63 ± 0.44 ^ef^	1.11 ± 0.11 ^b^	11.45 ± 1.75 ^c^
		MeOH	HBM	8.75 ± 0.67 ^ef^	11.50 ± 1.68 ^ef^	24.83 ± 0.87 ^h^	20.02 ± 0.13 ^g^	0.30 ± 0.06 ^e^	6.77 ± 0.98 ^d^
		Water	HBW	7.11 ± 0.66 ^f^	6.36 ± 0.42 ^gh^	19.67 ± 0.54 ^i^	18.18 ± 0.15 ^g^	0.38 ± 0.02 ^e^	11.44 ± 1.06 ^c^

* infusion method used to prepare water extracts; Values are reported as mean ± SD of three parallel measurements. PBD: phosphomolybdenum; MCA: metal chelating activity; TE: Trolox equivalent; EDTAE: EDTA equivalent. Different letters indicate significant differences in the tested extracts (*p* < 0.05).

**Table 4:** Enzyme inhibitory properties of extracts from aerial parts and bulb of *Allium lycaonicum*.

Extraction Methods	Parts	Solvents	Codes	AChE (mg GALAE/g)	BChE (mg GALAE/g)	Tyrosinase (mg KAE/g)	Amylase (mmol ACAE/g)	Glucosidase (mmol ACAE/g)
Maceration	Aerial parts	*n*-Hexane	MAH	4.36 ± 0.28 ^a^	20.99 ± 1.55 ^a^	127.35 ± 1.00 ^cd^	0.71 ± 0.02 ^ab^	3.01 ± 0.01 ^cd^
		MeOH	MAM	3.27 ± 0.09 ^b^	12.28 ± 0.61 ^f^	139.40 ± 0.32 ^a^	0.54 ± 0.02 ^cd^	2.53 ± 0.11 ^e^
		Water	MAW	1.47 ± 0.19 ^c^	5.83 ± 0.27 ^g^	30.15 ± 1.22 ^fg^	0.34 ± 0.01 ^e^	na
	Bulb	*n*-Hexane	MBH	4.22 ± 0.06 ^a^	17.53 ± 0.61 ^cd^	118.67 ± 1.52 ^e^	0.73 ± 0.03 ^a^	2.94 ± 0.02 ^cd^
		MeOH	MBM	4.29 ± 0.12 ^a^	15.25 ± 0.68 ^de^	132.39 ± 1.66 ^b^	0.51 ± 0.02 ^d^	3.25 ± 0.01 ^a^
		Water	MBW	1.12 ± 0.17 ^cd^	6.80 ± 0.67 ^g^	22.35 ± 1.49 ^h^	0.09 ± 0.01 ^f^	0.05 ± 0.02 ^g^
Soxhlet/infusion *	Aerial parts	*n*-Hexane	HAH	4.20 ± 0.27 ^a^	18.13 ± 0.62 ^bc^	129.86 ± 2.11 ^bc^	0.62 ± 0.09 ^bc^	3.03 ± 0.01 ^bcd^
		MeOH	HAM	3.08 ± 0.18 ^b^	13.01 ± 0.41 ^ef^	138.95 ± 1.07 ^a^	0.53 ± 0.01 ^cd^	3.07 ± 0.17 ^abc^
		Water	HAW	0.81 ± 0.03 ^d^	5.19 ± 0.67 ^g^	33.57 ± 1.14 ^f^	0.14 ± 0.01 ^f^	na
	Bulb	*n*-Hexane	HBH	4.75 ± 0.27 ^a^	20.36 ± 0.84 ^ab^	125.30 ± 1.53 ^d^	0.65 ± 0.03 ^ab^	2.86 ± 0.01 ^d^
		MeOH	HBM	4.59 ± 0.04 ^a^	18.48 ± 0.86 ^abc^	139.95 ± 0.22 ^a^	0.46 ± 0.03 ^d^	3.19 ± 0.05 ^ab^
		Water	HBW	1.14 ± 0.29 ^cd^	19.46 ± 1.99 ^abc^	27.40 ± 1.68 ^g^	0.12 ± 0.01 ^f^	0.48 ± 0.04 ^f^

* infusion method used to prepare water extracts; Values are reported as mean ± SD of three parallel measurements. GALAE: galantamine equivalent; KAE: kojic acid equivalent; ACAE: acarbose equivalent; na: not active. Different letters indicate significant differences in the tested extracts (*p* < 0.05).

## Data Availability

Data are contained within the article and Supplemental Materials.

## Supplementary Materials

The following supporting information can be downloaded at: https://www.mdpi.com/article/10.3390/foods12244507/s1, Table S1. HPLC–MS/MS acquisition parameters (dynamic-MRM mode) used for the analysis of the 38 marker compounds. References [18,49] was cited in Supplementary Materials.
